# CSFPre: Expressway key sections based on CEEMDAN-STSGCN-FCM during the holidays for traffic flow prediction

**DOI:** 10.1371/journal.pone.0283898

**Published:** 2023-04-05

**Authors:** Libiao Chen, Qiang Ren, Juncheng Zeng, Fumin Zou, Sheng Luo, Junshan Tian, Yue Xing

**Affiliations:** 1 Fujian Expressway Science & Technology Innovation Research Institute Co., Ltd., Fuzhou, Fujian, China; 2 Fujian Key Laboratory of Automotive Electronics and Electric Drive, Fujian University of Technology, Fuzhou, Fujian, China; 3 Research Institute for Transportation Big Data of Digital Fujian, Fuzhou, Fujian, China; University of Kurdistan Hewler, IRAQ

## Abstract

The implementation of the toll free during holidays makes a large number of traffic jams on the expressway. Real-time and accurate holiday traffic flow forecasts can assist the traffic management department to guide the diversion and reduce the expressway’s congestion. However, most of the current prediction methods focus on predicting traffic flow on ordinary working days or weekends. There are fewer studies for festivals and holidays traffic flow prediction, it is challenging to predict holiday traffic flow accurately because of its sudden and irregular characteristics. Therefore, we put forward a data-driven expressway traffic flow prediction model based on holidays. Firstly, Electronic Toll Collection (ETC) gantry data and toll data are preprocessed to realize data integrity and accuracy. Secondly, after Complete Ensemble Empirical Mode Decomposition with Adaptive Noise (CEEMDAN) processing, the preprocessed traffic flow is sorted into trend terms and random terms, and the spatial-temporal correlation and heterogeneity of each component are captured simultaneously using the Spatial-Temporal Synchronous Graph Convolutional Networks (STSGCN) model. Finally, the fluctuating traffic flow of holidays is predicted using Fluctuation Coefficient Method (FCM). Through experiments of real ETC gantry data and toll data in Fujian Province, this method is superior to all baseline methods and has achieved good results. It can provide reference for future public travel choices and further road network operation.

## Introduction

**Fig 1:**
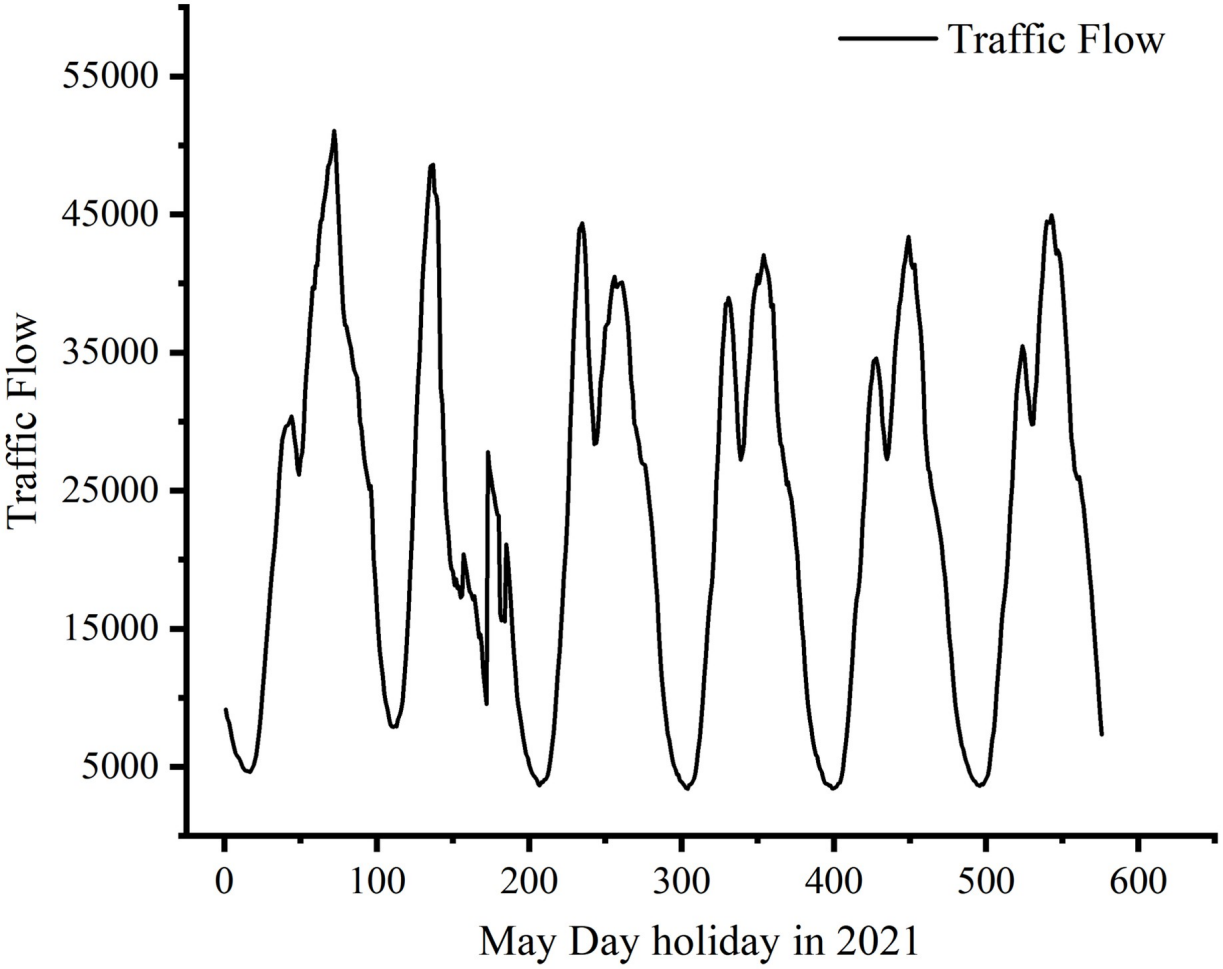
Traffic flow of Fuzhou-Xiamen expressway on May Day 2021.

At the end of 2020, the number of car ownership in Fujian Province had reached 7.313 million, and the mileage of expressways had breached 6,000 km, making China the 3rd ranked province for expressway road network density [1]. However, the growth rate of expressway traffic mileage is much lower than the growth rate of car ownership, which makes the contradiction between the supply and demand of expressways more serious, thus causing serious traffic congestion problems. The traffic flow during holidays contains more complex spatial-temporal characteristics, with a large range of changes, and the rules are very different from those on weekdays [2, 3], as shown in Fig 1. In addition, to provide convenience for people to travel, the State Council issued a notice on the toll free for the small-sized bus on expressways during major holidays (Spring Festival, Qingming Festival, Labor Day, and National Day) in 2012 [4], After the implementation of this policy, the national expressway traffic has increased drastically on major holidays, and traffic accidents and large-scale congestion have emerged, seriously affecting the quality of people’s travel. As soon as possible to solve the problem of free highway vehicle congestion on major holidays, it has become one of the key research directions in China’s traffic management. To further improve the operations efficiency of China’s expressways, 27,000 ETC gantries have been built in China, and 390 toll stations and 1,063 ETC main line physical gantries have been built in Fujian Province [5]. ETC gantry data and toll data can record the driving conditions of each vehicle on the expressway. Therefore, using ETC gantry data and toll data, we can accurately estimate the location of ETC gantries [6], prediction of traffic flow [7], travel time [8], travel speed [9], and service area vehicle recognition and dwell time estimation [10]. Since the expressway traffic management department cannot fully grasp the changing trend of road status during holidays, travelers cannot timely understand the changes in road conditions, resulting in too concentrated vehicle travel and difficulty in effective traffic control and diversion, causing traffic congestion [11]. Thus, how to accurately predict the traffic flow during holidays and reduce the occurrence of traffic congestion is an important challenge to realizing the fine management of expressways [12].

Aiming at this challenge, some scholars have been researching the expressway traffic flow during holidays. For example, Liu and Dai proposed a method based on deep learning that uses BP neural network models and wavelet filtering techniques to predict holiday traffic flow [13]. Since the Genetic Algorithm (GA) can effectively improve the efficiency and feasibility of the algorithm, Chen et al. proposed an improved GA-BP to predict holiday traffic flow [14]. In addition, Ji and Ge analyzed the spatial and temporal characteristics of holiday traffic flow and proposed a deep learning model based on Long Short-Term Memory neural network-Support Vector Regression (LSTM-SVR) for holiday traffic flow prediction [15]. The establishment of these prediction models makes up the blank of holiday expressway traffic flow prediction. But there are several shortcomings as follows. Firstly, most of the research is vehicle detector data, and vehicle detectors are placed at a specific location to report the traffic flow at those specific locations, and the destruction rate is high and the precision is low. Secondly, most models ignore the fact that traffic flow data may include a large amount of noise and use separate components to capture spatial and temporal correlations, not considering the direct capture of local spatial-temporal correlations. Finally, the limited number of holiday traffic flow samples is ignored. Therefore, this paper studies how to extract the salient features of traffic flow under limited data conditions, and capture these complex local spatial-temporal correlations and heterogeneities at the same time, to accurately predict the traffic flow on holidays.

In this paper, a holiday traffic flow prediction model is established. Firstly, the ETC gantry data and toll data are self-adaptive processed by fusing the CEEMDAN algorithm and decomposed into multiple scales of Intrinsic Mode Functions (IMFs). Secondly, the STSGCN model is used to predict each IMF, and the local spatial-temporal correlation of traffic flow data is fully explored. Finally, the FCM method is used to predict the traffic flow in the fluctuating part of holidays, which solves the problem of lack of traffic flow data during holidays in the past. By experiments on holiday expressway ETC data and toll data from Fuzhou to Xiamen in Fujian Province, the holiday traffic flow prediction model is verified to have good prediction performance. As the following is an overview of the main contributions of this paper: (1) A new method is proposed to predict traffic flow on key sections of expressways during holidays by using ETC gantry data and toll data; (2) The holiday traffic flow prediction model is composed of CEEMDAN, STSGCN and FCM. The model can well extract the uncertainty and nonlinear characteristics of traffic flow. A localized spatial-temporal graph is established, which only considers the spatial-temporal correlation of the ETC gantry and toll station associated with the target section. FCM is used to predict the part of the holiday traffic flow volatility; (3) By conducting extensive experiments on real holiday ETC gantry datasets and toll datasets, we verified that our proposed prediction method outperforms all baseline methods.

The rest of the paper is organized as follows: The rest of the paper is organized as follows: Section II reviews the literature on expressway traffic flow forecasting. Section III presents the problem definition of holiday traffic flow prediction. Section IV introduces the proposed models and methods. Section V conducts a detailed experiment on the developed model, and presents the experimental results as well as the prediction performance of the proposed model. Finally, our conclusions are presented.

## Related work

At present, expressway traffic flow prediction is mainly classified into two categories: one is a regression model based on mathematical statistics, and the other is a machine learning model. In traffic flow forecasting, the most commonly used Autoregressive Integrated Moving Average model (ARIMA) and its variants [16–18]. Yao et al. [19] proposed a hybrid model of ARIMA and Generalized Autoregressive Conditional Heteroscedasticity (GARCH) for short-term traffic flow prediction. Lu [20] proposed a method for combined short-time traffic flow prediction based on the ARIMA model and LSTM neural network, which makes use of ARIMA and LSTM to obtain linear and nonlinear features of traffic data, respectively, using the dynamic weighting of sliding windows, and finally combining the prediction effects of these two techniques. Zhang et al. [21] proposed a wavelet transform based on seasonal ARIMA and combined it with external factors to predict traffic flow. However, how to capture the temporal and spatial correlations under nonlinear relationships is the core problem of traffic flow prediction. In general, traffic flow data are usually nonlinear, and ARIMA can only capture linear relationships by nature and cannot reflect nonlinear relationships and temporal correlation.

In capturing nonlinearities and temporal correlations, machine learning models have shown excellent performance. For example, Support Vector Machine (SVM) [22] and random forest [7] adequately capture nonlinear features and temporal characteristics. Lin et al. [23] proposed a new spatial time delays traffic sequences screening algorithm based on maximum information coefficients. On this basis, the SVR method and k-nearest neighbor algorithm are combined to transform the selected time delays traffic sequence into a traffic state vector to predict traffic flow. Based on the SVR algorithm, Zou et al. [24] established a prediction model by sliding window, and optimized the SVR model by Particle Swarm Optimization (PSO). However, most of these models only consider the temporal correlation and do not adequately consider the spatial correlation of traffic flow.

In recent years, as an important branch of machine learning, deep learning models have been widely applied in the field of traffic flow prediction. Yang et al. [25] proposed a multi-feature fusion framework that combines a Convolutional Neural Network (CNN) and external factors (weather and holidays) for traffic flow prediction. Qiao et al. [26] proposed a method that uses one-dimensional convolution neural network (1DCNN) for spatial information extraction and used LSTM to obtain temporal information of traffic data. To efficiently make use of the spatial-temporal dependency, a Graph Convolutional Neural (GCN) network is proposed to make up for the deficiencies of the CNN model. Zhao et al. [27] proposed a temporal graph convolutional network using a combination of GCN and Gated Recurrent Unit (GRU) to obtain the spatial-temporal characteristics of traffic flow. Yu et al. [28] proposed a Spatial-temporal Graph Convolutional Network (STGCN) with multiple graph convolutional layers to capture spatial features and one-dimensional convolution to fit temporal features. Guo et al. [29] increased the spatial-temporal attention mechanism on this basis, which can capture dynamic spatial-temporal information in traffic data adaptively. Whether on weekdays or weekends, these traffic flow prediction models have good results. However, the traffic flow during holidays is highly irregular and volatile, making these models unable to adequately capture the holiday characteristics, which affects the validity of the prediction. Therefore, how to further improve the accuracy of expressway traffic flow prediction during holidays still is meaningful research.

To accurately predict holiday expressway traffic flow, many scholars have conducted research. Xie et al. [30] used improved back propagation neural network (IGA-BPNN) optimized by GA to develop non-holiday and holiday traffic flow prediction models, and achieved accurate prediction. Lu et al. [31] analyzed the holiday traffic flow characteristics, divided the holiday traffic flow into regular and fluctuating parts, and predicted the regular flow by LSTM and the fluctuating flow by FCM. Luo et al. [32] proposed a hybrid prediction method combining the Discrete Fourier Transform (DFT) and SVR. Zhang et al. [33] proposed the use of CNN, GRU, and Convolutional LSTM (ConvLSTM) networks to analyze the temporal and spatial characteristics of holiday traffic flow.

The above research mainly focuses on the regularity of holiday traffic flow. But due to the limited sample volume of historical traffic flow during holidays, the specific patterns cannot be well modeled, and there are obvious differences in the flow data of the same period in different years, especially in years, which are far apart, so the accuracy of prediction cannot be ensured. Improve prediction accuracy by using ETC portal data and toll station data while capturing their temporal and spatial correlation. In this paper, we propose a method of ETC gantry data and toll data to predict the traffic flow of expressways during holidays.

## Problem defintion

In this section, the relevant definitions are given. We first define some critical parameters, and then propose the problem of traffic flow prediction on the key sections during expressway holidays. The purpose of this research is to use historical ETC gantry data and toll data to predict the traffic flow in the target section at the next time interval. The traffic flow data extracted from the historical ETC gantry data and toll data are counted in a time series with a time interval of 15 minutes.

Definition 1 (Expressway *QD*): Each ETC gantry and toll station on the expressway is generically referred to as a *Node*, and two adjacent nodes on the road constitute an expressway section *QD*, *QD* = (*Q*, *Distance*), *Q* = (*Node_1_*, *Time_1_*, *Node_2_*, *Time_2_*), where *Node_1_* is the start of the section, *Node_2_* is the end of the section, and Distance is the actual distance of the section.

Definition 2 (Trajectory of a vehicle *Traj*): The sequence of nodes formed by a vehicle entering the toll station, passing through the ETC gantry, and finally exiting from the toll station is called *Traj* = (*Node_1_*, …, *Node_N_*), where *Node_1_* is called the starting point of the trajectory, *Node_N_* is called the end point of the trajectory.

Definition 3 (The expressway network *G*): In order to express the spatial topology of each ETC gantry and toll station in the whole expressway network, the graph *G* = (*V*, *E*, *A*) is defined, where *V* = (*Node_1_*, …, *Node_N_*) denotes the set of all nodes on the expressway, *N* denotes the number of nodes, *E* denotes the set of edges connected between nodes. *A* is the adjacency matrix of road network *G*.

Definition 4 (Traffic flow matrix $X_{G}^{t}\in R^{N\times P}$): Where *P* is the number of node attribute features, the length of the traffic flow history time series, and *t* denotes the time step. This matrix represents the observed values of the expressway road network *G* at time *t*.

Therefore, the traffic flow prediction problem for the entire key section of the expressway during holidays can be formulated as follows. Learn a mapping function *F* that maps historical traffic flow spatial-temporal data ($X_{G}^{t-n}$, …, $X_{G}^{t-1}$, $X_{G}^{t}$) to future observations $X_{G}^{t+1}$ in the expressway road network *G*, and *T* denotes the length of the historical traffic flow time series. Therefore, the problem of predicting the traffic flow for the next time interval *t+1* in the target segment can be defined as:

$$\begin{array}{c} X_{G}^{t+1}=F\left(\left(X_{G}^{t-n},\ldots ,X_{G}^{t-1},X_{G}^{t}\right),G\right) \end{array}$$ (1)

## Methodology

In this section, we introduce in detail our proposed method for forecasting traffic flow on key sections of expressways based on holidays, which combines holiday traffic flow forecasting and holiday traffic flow fluctuation forecasting. First, the basic framework of traffic flow forecasting on the holiday key sections is introduced. Then, the four components of data preprocessing, CEEMDAN, STSGCN, and FCM will be introduced separately.

### Overview of the overall framework

**Fig 2:**
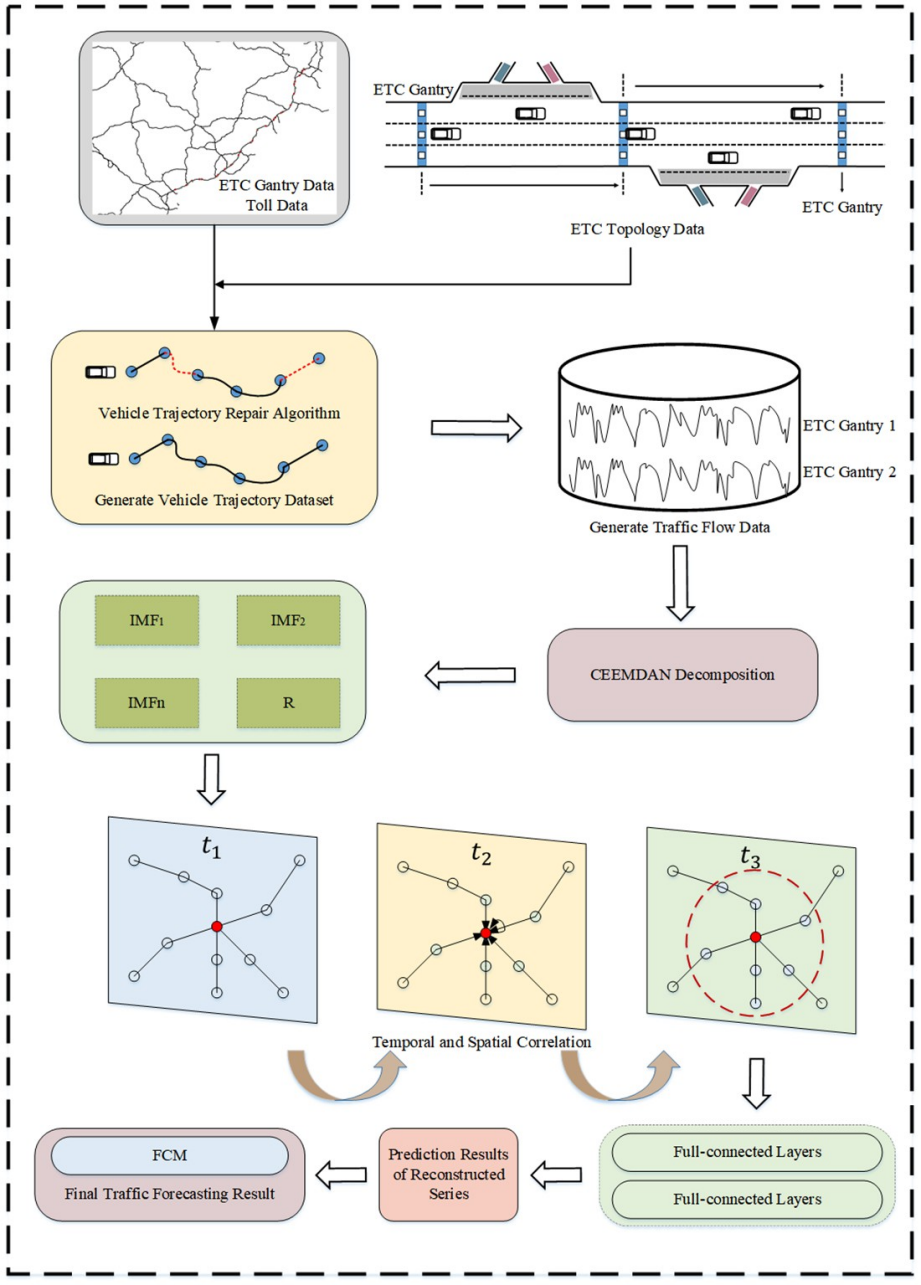
Summary of forecasting models.

Expressway traffic flow data during holidays are more random and volatile, which makes prediction more difficult. In this paper, we propose a method for predicting holiday expressway traffic flow based on ETC gantry data and toll data, as shown in Fig 2. First, data need to be preprocessed to ensure its accuracy. On this basis, based on the time series, the vehicle trajectory is constructed based on the gantry topology data, and for the missed vehicle trajectory, the vehicle trajectory repair algorithm is utilized to repair the trajectory and ensure the completeness of the data. The nonlinearity and uncertainty of holiday traffic flow are more prominent. The CEEMDAN algorithm is applied to add adaptive positive and negative Gaussian white noise to the time series components of holiday traffic flow, thereby realizing the adaptive processing of holiday traffic flow. At the same time, due to the limitation of the spatial distance between the ETC gantry and the time range of the traffic flow sequence, we construct a localized spatial-temporal graph to represent the spatial-temporal correlations between the gantry and the toll station related to the target gantry after 15 minutes and use the STSGCN [34] model to extract the spatial-temporal features of the localized spatial-temporal graph. The decomposed IMF are predicted by the STSGCN model, the spatial-temporal characteristics of the traffic flow data are fully explored, and the predicted values of each IMF component are superimposed as the prediction results of the STSGCN model. During the holidays, the characteristics of the weekday traffic flow will be broken, and the traffic flow in a certain period appears to be a continuous peak, and we use FCM to predict the fluctuating flow. Finally, we can get the predicted holiday traffic flow.

### Data preprocessing

ETC gantry data and charging data acquisition process, due to equipment abnormal, wireless crosstalk, bad weather, and other uncontrollable random factors, resulting in the gathered data mainly exist in the following three kinds of abnormal problems [35]. (1) Data duplication: This is usually due to network or system faults or software vulnerabilities, data is repeatedly uploaded, resulting in duplicate transaction records. (2) Data missing: The problem that data cannot be efficiently collected appears. This is usually due to traffic congestion, the ETC gantry failing to identify the vehicle or the device being abnormal and other reasons. (3) Data error: Data records do not match with normal traffic regulations, such as the entrance toll station time being later than the ETC gantry transaction time, and the vehicle is detected by the reverse gantry. These abnormal data seriously affect the application value of data mining. To reduce the impact of errors on the accuracy of the established prediction model, this type of data will first be removed.

**Fig 3:**
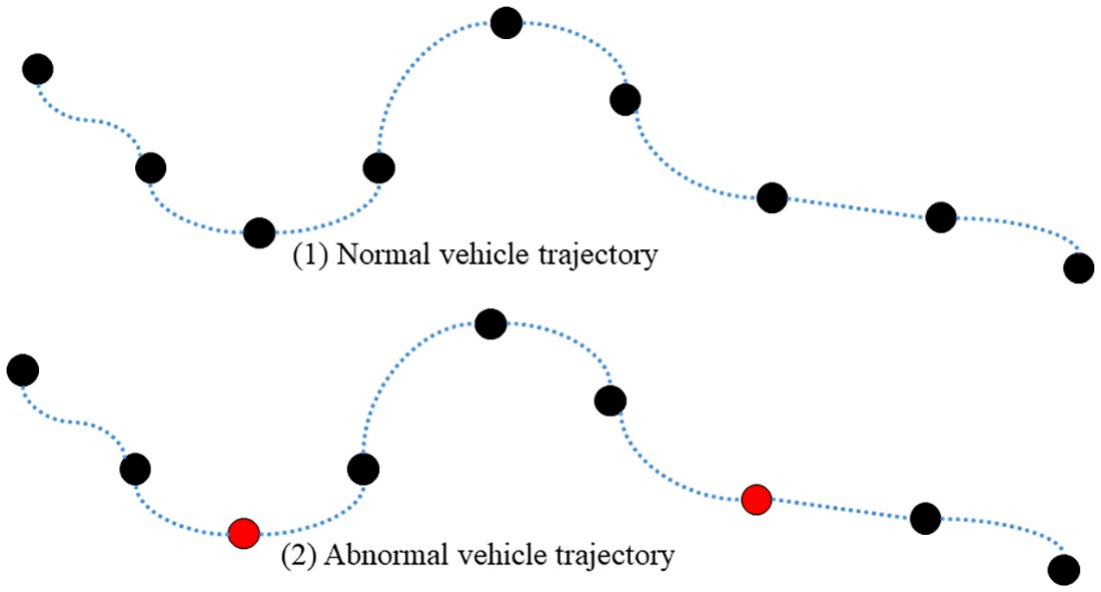
Vehicle trajectory diagram.

Vehicle trajectory repair algorithm: the abnormal data of ETC gantry transaction data is removed, and the vehicle trajectory data suffer from different degrees of missing, as shown in Fig 3, the black color part represents this ETC gantry data is recorded normally, and the red color part represents the missing of this ETC gantry data. If the vehicle trajectory is not repaired, when the traffic flow of each section is counted, some vehicles will not be counted effectively, which will have some effect on the final prediction results. Therefore, it is necessary to implement vehicle trajectory repair to improve the reliability of prediction.

According to the time series, the driving trajectory of each vehicle is constructed, the ETC gantry search is applied to the driving trajectory of each vehicle by using the expressway road network *G*, and the two adjacent ETC gantries are rifled through to check whether the two adjacent gantry topologies exist in the expressway road network *G*. If not, based on these two gantries for road search, interpolation vehicle trajectory. The average speed of the vehicle passing through the two gantries is calculated, and this average speed is regarded as the average speed of all the sections between these two gantries. According to the distance between the adjacent gantries, the travel time of each section can be calculated, so that the vehicle travel trajectory can be repaired.

### CEEMDAN

The ETC gantry system is susceptible to external factors, the gathered data will contain more noise. EMD is a classical adaptive method to solve nonlinear and non-smooth signal problems based on noise frequency processing. It does not need to set any basis function in advance, and only relies on its time scale characteristics to decompose the signal to obtain IMF with different amplitudes and a residue (res) [36]. However, the IMF components obtained by EMD have mode mixing, and the appearance of a mixing problem causes inaccurate time-frequency distribution, which makes some IMFs lose their physical meaning. EEMD [37] added the Gaussian white noise to the original signal to solve the mode mixing problem. But the Gaussian white noise after decomposition cannot be eliminated, and the completeness of the decomposition is poor, resulting in large reconstruction errors in the algorithm. To overcome these problems, Torres [38] proposed CEEMDAN, which improves EEMD by adding adaptive Gaussian white noise to each decomposition process to improve the completeness of the decomposition of EEMD, reduces the reconstruction error, have the highest decomposition efficiency and reduce the computational cost greatly.

The implementation steps of traffic flow time series data based on CEEMDAN decomposition are as follows. First, the time series data of the original traffic flow *y*(*t*) is denoted, *IMF_k_* is the kth order IMF obtained after EMD decomposition, *v*^*j*^(*t*) is the Gaussian white noise signal satisfying the standard normal distribution added at time *j* (*j* = 1,2,…N) during the decomposition process, and *ε*_*j*_ is the signal-to-noise ratio at each stage of the decomposition of the original traffic flow time series data *y*(*t*).

(1) Adding the Gaussian white noise *v*^*j*^(*t*) to the original traffic flow time series data *y*(*t*) at the jth, the traffic flow time series can be expressed as:

$$\begin{array}{c} y^{j}\left(t\right)=y\left(t\right)+\varepsilon_{j}v^{j}\left(t\right) \end{array}$$ (2)

(2) The EMD method is used to decompose the traffic flow data and obtain the *1*st order modal component as $IMF_{1}^{j}\left(t\right)$, the mean value as *IMF*_1_(*t*) and the 1st order residual component as *res*_1_(*t*).

$$\begin{array}{c} {\text{IMF}}_{1}\left(t\right)=y\left(t\right)-{\text{IMF}}_{1}^{j}\left(t\right) \end{array}$$ (3)

$$\begin{array}{c} {\text{res}}_{1}\left(t\right)=y\left(t\right)-{\text{IMF}}_{1}\left(t\right) \end{array}$$ (4)

(3) The *2*nd order modal component as $IMF_{2}^{j}\left(t\right)$ and the *2*nd order residual component sequence as *res*_2_(*t*).

$$\begin{array}{c} {\text{IMF}}_{2}\left(t\right)=\frac{1}{N}\sum_{j=1}^{N}E_{1}\left(r_{1}\left(t\right)+\varepsilon_{1}E_{1}\left(v^{j}\left(t\right)\right)\right) \end{array}$$ (5)

$$\begin{array}{c} {\text{res}}_{2}\left(t\right)={\text{res}}_{1}\left(t\right)-{\text{IMF}}_{2}\left(t\right) \end{array}$$ (6)

(4) The *k*th order residual component as *res*_*k*_(*t*) and the *k+1*th order modal component as $IMF_{k+1}^{j}\left(t\right)$.

$$\begin{array}{c} {\text{res}}_{k}\left(t\right)=r_{k-1}-{\text{IMF}}_{k}\left(t\right) \end{array}$$ (7)

$$\begin{array}{c} {\text{IMF}}_{k+1}\left(t\right)=\frac{1}{N}\sum_{j=1}^{N}E_{1}\left(r_{k}\left(t\right)+\varepsilon_{k}E_{k}\left(v^{j}\left(t\right)\right)\right) \end{array}$$ (8)

(5) Repeat the above steps until the residual component cannot continue to be decomposed (monotonic function or no more than two extreme points), and the final residual component can be expressed as:

$$\begin{array}{c} \text{res}\left(t\right)=y\left(t\right)-\sum_{k=1}^{N}{\text{IMF}}_{k}\left(t\right) \end{array}$$ (9)

The IMFs together constitute the characteristics of the original signal on different time scales. The residual components clearly show the trend of the original traffic flow time series and effectively reduce the prediction error.

### STSGCN

**Fig 4:**
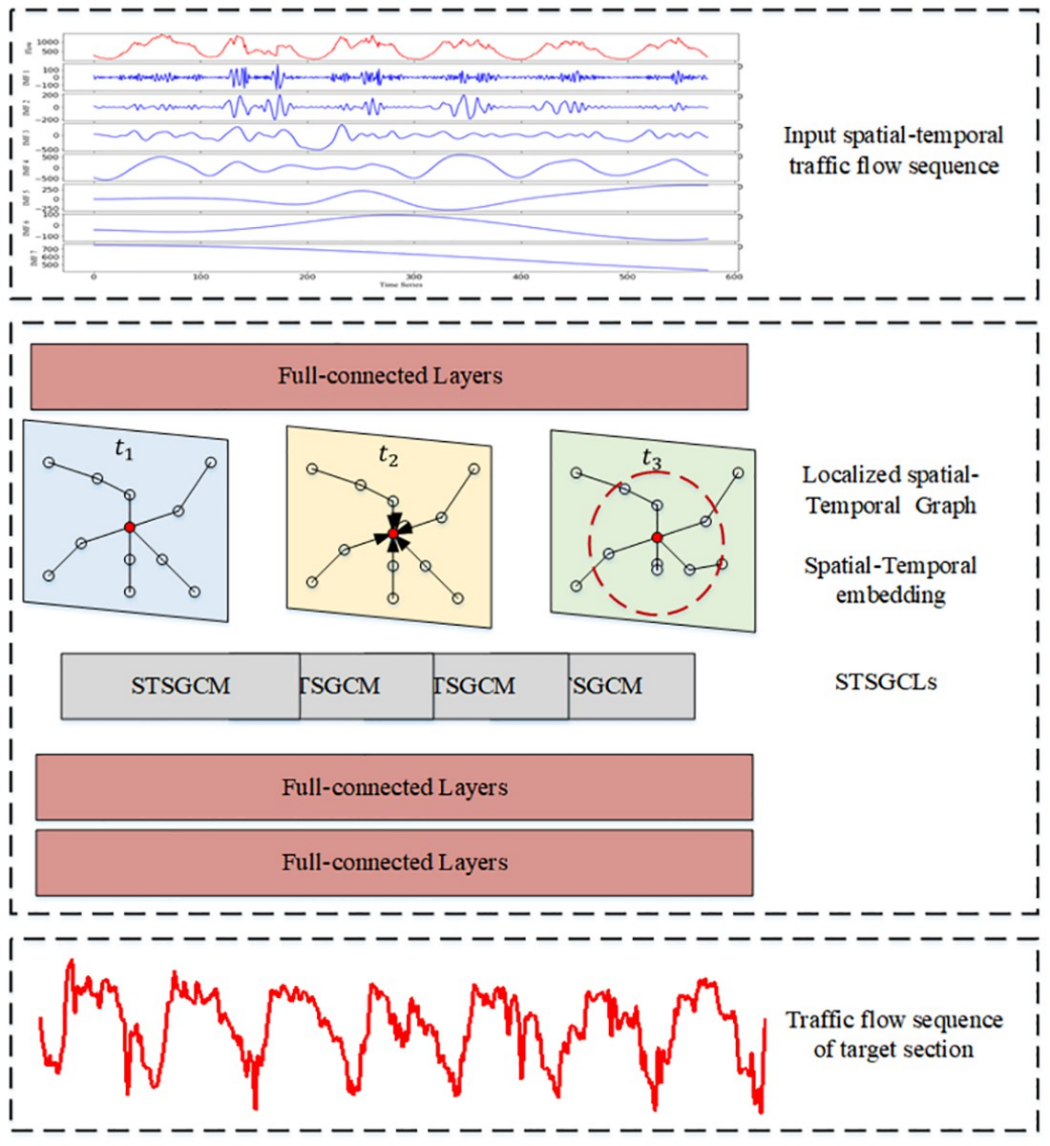
STSGCN architecture.

The STSGCN model uses an end-to-end approach to take historical traffic flow spatial-temporal sequence data as input and output it for future traffic flow spatial-temporal sequence data. First, the spatial-temporal sequence information of historical traffic flow is input to a fully connected layer, which is designed to map from low-dimensional traffic flow features to high-dimensional dimensions. Then, the Spatial-Temporal Synchronous Graph Convolutional Layer (STSGCL) automatically divides the localized spatial-temporal graph and the traffic flow matrix, obtains the spatial-temporal correlation of each l localized spatial-temporal graph, and aggregates and crops the output of the convolution module for each spatial-temporal graph. The STSGCN framework is shown in Fig 4.

#### Input layer

**Fig 5:**
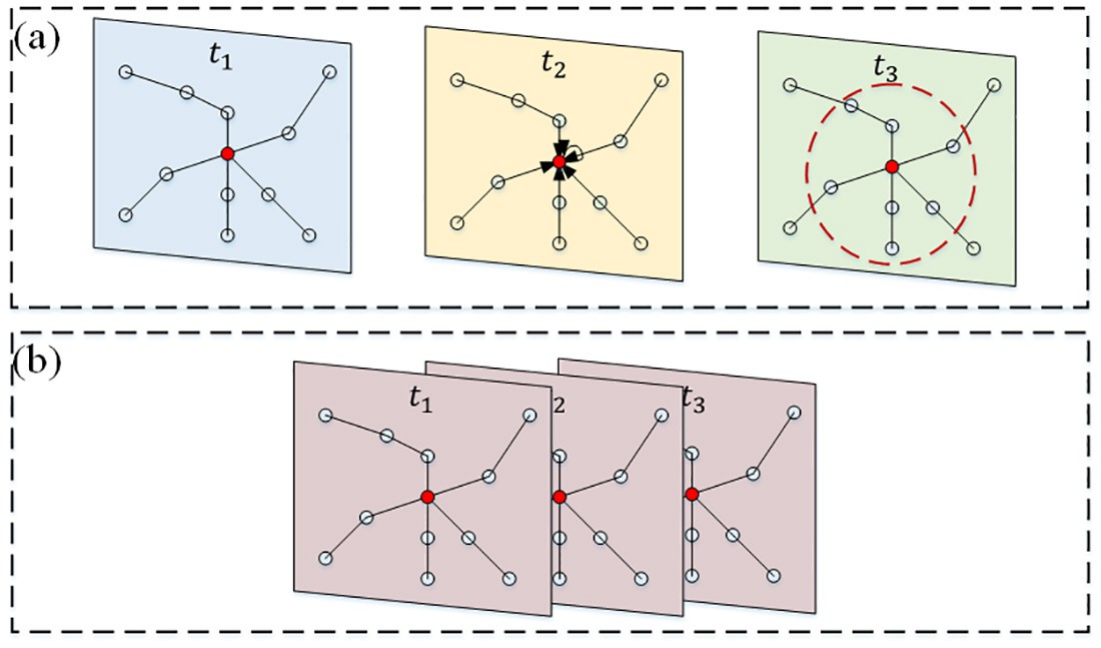
Spatial-temporal graph representation. (a) Spatial graph; (b) Localized spatial-temporal graph.

The input traffic flow spatial-temporal sequence data *X*_*G*_ ∈ *R*^*N*×*T*^ is converted to *X*_*G*_ ∈ *R*^*N*×*P*×*T*^ by a fully connected layer. To directly capture the influence of the target segments on their adjacent segments, each segment is connected to its segment at two time points before and after to construct a spatial graph containing three times before, during, and after, which is called the localized spatial-temporal graph [39]. The adjacency matrix of the spatial-temporal graph is expressed by *A* ∈ *R*^*N*×*N*^, and the adjacency matrix of the local spatial-temporal graph constructed using 3 consecutive spatial-temporal graphs can be expressed by $A^{{}^{\prime }}\in R^{3N\times 3N}$. For ETC gantries *i* in the spatial graph, the new index of the localized spatial-temporal graph is calculated by (*t* − 1)*N* + *i* (0 ≤ *t* ≤ 3). The spatial connectivity of the nodes is the same in each time step *t*. If two ETC gantries are interconnected in this localized spatial-temporal graph, the corresponding value in the adjacency matrix is set to 1. Fig 5(b) represents the related localized spatial-temporal graph extended from the spatial graph in (a).

Putting nodes with different time steps in the localized spatial-temporal graph will blur the spatial-temporal properties of each node. Aiming at the traffic flow spatial-temporal sequence data *X*_*G*_ ∈ *R*^*N*×*T*^, a learnable temporal embedding matrix *T*_*e*_ = *R*^*P*×*T*^ and a spatial embedding matrix *S*_*e*_ = *R*^*N*×*P*^ are established for the extraction of spatial-temporal features. To enhance the modeling capability of spatial-temporal correlation, these two embedding matrices are added to the spatial-temporal sequence with broadcast operation [40]. Thus, the new representation of traffic flow spatial-temporal sequence data is:

$$\begin{array}{c} {X_{G}}^{{}^{\prime }}=X_{G}+T_{e}+S_{e}\in X_{G}\in R^{N\times P\times T} \end{array}$$ (10)

#### Spatial-temporal synchronous graph convolutional module (STSGCM)

**Fig 6:**
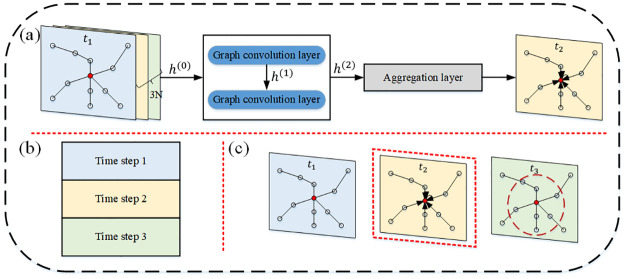
STSGCM architecture. (a) STSGCM; (b) Aggregation operation; (c) Cropping operation.

A spatial-temporal synchronous graph convolutional module is constructed to obtain local spatial-temporal features for the traffic flow spatial-temporal sequence data ${X_{G}}^{{}^{\prime }}$, which contains two graph convolutional layers and one aggregation layer, as in Fig 6(a). The adjacency matrix $A^{{}^{\prime }}$ determines the size of the aggregated weights. If the adjacency matrix $A^{{}^{\prime }}$ of the localized spatial-temporal graph contains only 0 and 1, when the graph convolution operation is applied, even if there are topological relationships between ETC gantries, their features will be aggregated when there is no correlation at some time node. To enhance the performance of spatial-temporal feature extraction, a learnable mask matrix *W*_*mask*_ ∈ *R*^3*N*×3*N*^ is embedded in the adjacency matrix $A^{{}^{\prime }}$, the mask matrix *W*_*mask*_ ∈ *R*^3*N*×3*N*^ and the adjacency matrix $A^{{}^{\prime }}$ are multiplied to generate the weighted localized spatial-temporal graph adjacency matrix $A_{mask}^{{}^{\prime }}$. The calculation formula is as follows.

$$\begin{array}{c} A_{\text{mask}}^{{}^{\prime }}=W_{\text{mask}}⊗A^{{}^{\prime }} \end{array}$$ (11)

Currently, there are two main types of graph convolutional neural networks, one based on the spatial domain [41] and the other based on the frequency domain [42]. In this paper, we use GLU as the activation function to perform graph convolution operations based on the spatial domain graph convolution method. The purpose is to aggregate the information related to the target gantry and the surrounding neighbor nodes through the defined aggregation function to update the characteristics of the traffic flow through the target gantry. This graph convolution operation can be expressed by the Eq (12).

$$\begin{array}{c} h^{\left(l\right)}=\sigma \left(A_{\text{mask}}^{{}^{\prime }}h^{\left(l-1\right)}W+b\right) \end{array}$$ (12)

where *h*^(*l*)^ represents the output of the *l*th GCN layer, *σ* represents the GLU activation function, $A_{mask}^{{}^{\prime }}$ epresents the weighted localized spatial-temporal graph adjacency matrix, *h*^(*l*−1)^ represents the input of the *l*th GCN layer and the output of the *l-1*th GCN layer, *W* and *b* are parameters.

To capture the spatial-temporal correlation of ETC gantries over a wider range, multiple stacked spatial-temporal synchronous graph convolution operations are designed to expand the region of their feature aggregation. For the multi-layer STSGCM, the output of each graph convolution operation is input into the aggregation layer for aggregation, as in Fig 6(b); on the basis of it, the contents of the previous and the next time steps are deleted, and the aggregated features of the intermediate time steps are retained, as in Fig 6(c).

There are two operational steps in the aggregation layer: the aggregation operation and the cropping operation. In the aggregation operation, max-pooling is used to maximize all the outputs of the graph convolution layer to obtain the aggregated representation of the intermediate spatial-temporal information. The max-pooling *h*_*Agg*_ = *max*(*h*^1^, *h*^2^, …, *h*^*l*^) in the aggregation operation. Then by the clipping operation, only the intermediate time aggregated features are kept. The redundancy in the model is reduced and the generalization ability of the model is improved.

#### STSGCL

The aim is to predict the traffic flow of the target section in the next 15 minutes. Therefore, a local spatial-temporal graph based method is proposed, which can capture the local spatial-temporal characteristics of the traffic flow for the before and after 15 minutes. Since spatial-temporal sequence data of longer time ranges are input in the actual situation, the traffic flow has different spatial-temporal dependencies in different times and spaces. Therefore, STSGCL is constructed using sliding window and multi-component modeling to deal with the spatial-temporal heterogeneity in spatial-temporal network sequences, the long-distance spatial-temporal features of traffic flow spatial-temporal sequence data are extracted.

For the traffic flow spatial-temporal series data of time length *T*, *T-2* time segments can be cut out by sliding windows. The independent localized spatial-temporal graphs and the corresponding traffic flow matrix are then constructed on each of the *T-2* time segments. They are input to *T-2* STSGCM to capture the local spatial-temporal correlation. The outputs of the *T-2* STSGCM are combined to form a new spatial-temporal sequence of traffic flows, and the output *M* of the STSGCL is expressed as:

$$\begin{array}{c} M=[M_{1},M_{2},\ldots ,M_{T-2}] \end{array}$$ (13)

Where *M*_*i*_ represents the output of the *i*th STSGCM and *T* represents the time step of the STSGCL input.

#### Output layer

An output layer is deployed after the STSGCL, which consists of two fully connected layers to map the final output to the target sequence. It is first transposed, then its dimensions are reorganized and put into two fully connected layers. The output is $X_{G}^{t+1}=\left[X_{G}^{t-n},\ldots ,X_{G}^{t-1},X_{G}^{t}\right]$.

## FCM

Fluctuation coefficients of traffic flow at different times of holidays are directly related to the fluctuation coefficients of historical traffic flows and also related to the prediction coefficients of traffic flow changes at that time of the month. Therefore, the traffic flow during the holiday period can be considered as two parts: the weekday traffic flow and the fluctuating traffic flow based on the weekday traffic flow.

$$\begin{array}{c} X_{G(H)}^{t+1}=\alpha \cdot X_{G(R)}^{t+1} \end{array}$$ (14)

Where $X_{G(H)}^{t+1}$ is the traffic flow at the next moment of the holiday, $X_{G(R)}^{t+1}$ is the traffic flow at the next moment of the weekday, and *α* indicates the rate of the average traffic flow between the current moment of the holiday and the corresponding moment of the month.

## Experiments

In this section, we use a real dataset of the Fuzhou-Xiamen holiday expressway to validate the feasibility of the expressway holiday traffic flow prediction model. We first introduce the dataset used in our study and then describe the setup and evaluation metrics of our model. We also select several convention models as baseline models to compare the predictive performance. Finally, the experimental results are analyzed from several angles.

### Data

This study is based on the Fuzhou-Xiamen expressway road network in Fujian Province in 2021. The data are mainly divided into two types of data, one is the ETC gantry data and toll data from April 30 to May 5, 2021 of Fujian Expressway Science and Technology Innovation Research Institute Co., Ltd., the main attributes are shown in Tables 1 and 2. The second is the expressway road network topology data, which includes the connection relationship and actual distance between each ETC gantry, ETC gantry and toll station.

**Table 1:** ETC gantry system partial transaction data attribute table.

Attribute name	Examples	Attribute name	Examples
Trade ID	3408119***2698	OBU Sn	00361***96BT7
Trade Time	2021/5/1 18:27:51	Vehicle Class	1
Flag ID	350A23	Enter Time	2021/5/1 18:21:35
User Type	0	Enter Station	3506
Flag Type	0	OBU ID	67B***56E

**Table 2:** Toll system partial transaction data attribute tablee.

Attribute name	Examples	Attribute name	Examples
Enter Time	2021/5/1 07:14:30	Enter Station	2902
Exit time	2021/5/1 07:18:271	Exit Station	2901
Trade ID	340119***2698	Card Type	23
User Type	0	Spend Time	237
Vehicle Class	1	OBU ID	164***226

The ETC gantry data is matched with the toll data to get the trajectory of each vehicle. There exist incidents of missing transactions and wrong transactions because of the expressway ETC system. For example, when a vehicle passes through the gantry of the upstream section, it is missed transaction due to the blocking of a larger vehicle in front of it, or it is too close to the gantry of the downstream section causing a wrong transaction. On this basis, a vehicle trajectory repair algorithm is proposed for trajectory repair to complement the integrity of the data set. Then the traffic flow dataset of each ETC gantry and toll station is counted at 15-minute intervals, as shown in Table 3. The z-score is used for global normalization, and the normalization formula is shown in Eq (15).

$$\begin{array}{c} Z=\frac{X-X_{\text{mean}}}{X_{\text{std}}} \end{array}$$ (15)

*Z* represents the normalized data, *X*, *X*_*mean*_, and *X*_*std*_ represent the initial value, the mean and standard deviation of the historical time series traffic flow, respectively.

**Table 3:** Flow statistics table for each gantry and toll station.

Gantry number	341601	A3304	…	340249	3106
Time					
00:00-00:15	0	26	…	22	7
00:15-00:30	195	43	…	55	15
⋮	⋮	⋮	⋱	⋮	⋮
23:30-23:45	206	30	…	50	30
23:45-00:00	104	25	…	66	25

### Evaluation metrics and experimental setup

To evaluate the prediction performance of different models, the experiments use Mean Absolute Error (MAE), Mean Absolute Percentage Error (MAPE), and Root Mean Square Error (RMSE) as evaluation metrics. And Huber loss is used as the loss function. Compared with the common loss function, Huber loss is less sensitive to outliers and improves the speed of training.

$$\begin{array}{c} \text{MAE}=\frac{1}{N}\sum_{i=1}^{N}|y_{i}-{\hat{y}}_{i}| \end{array}$$ (16)

$$\begin{array}{c} \text{MAPE}=\frac{1}{N}\sum_{i=1}^{N}|\frac{y_{i}-{\hat{y}}_{i}}{y_{i}}| \end{array}$$ (17)

$$\begin{array}{c} \text{RMSE}=\sqrt{\frac{1}{N}\sum_{i=1}^{N}{\left(y_{i}-{\hat{y}}_{i}\right)}^{2}} \end{array}$$ (18)

where *y*_*i*_ is the actual traffic flow, ${\hat{y}}_{i}$ is the predicted traffic flow, and *N* is the number of ETC gantries and toll stations.

**Fig 7:**
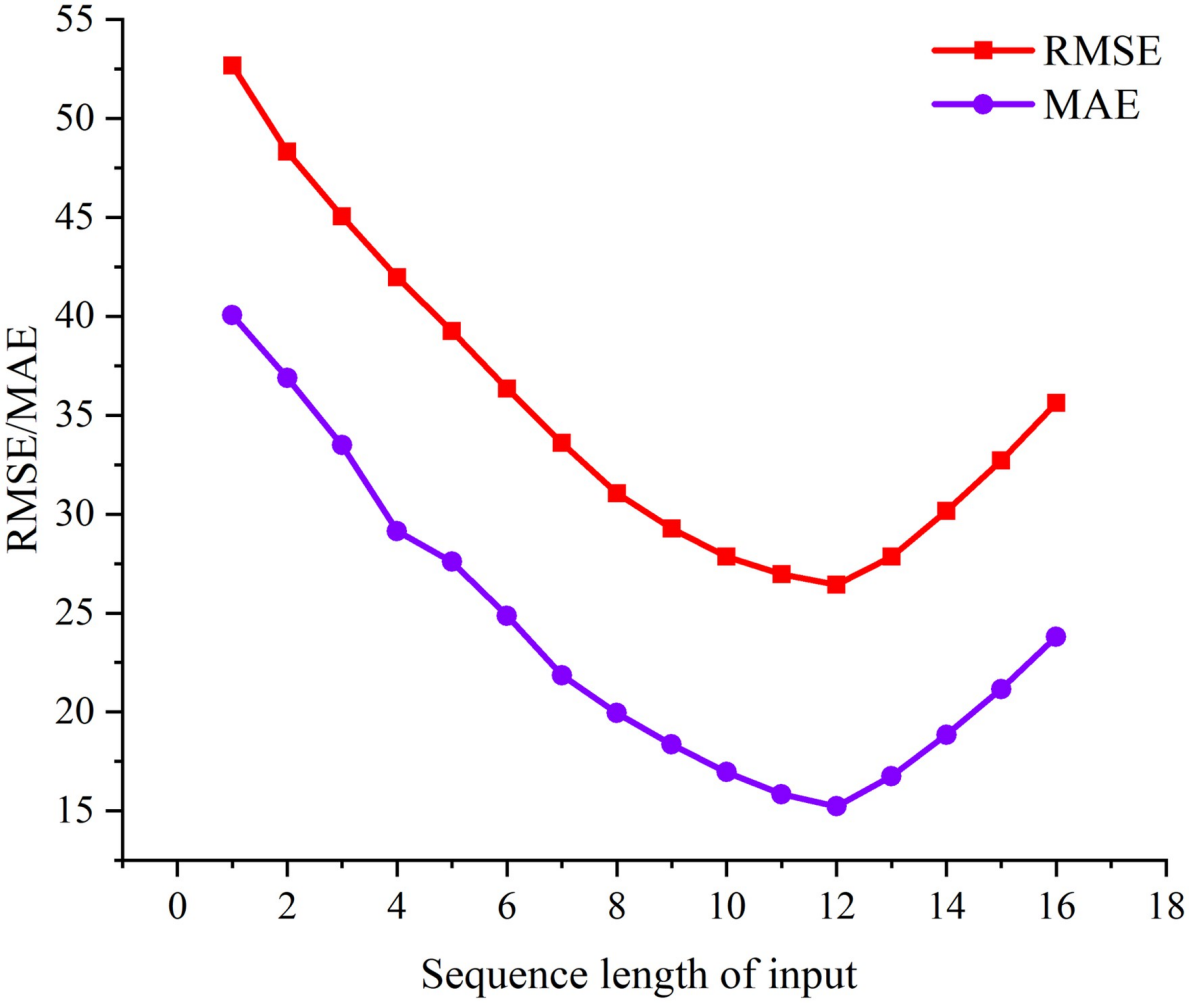
Analysis of sequences of different lengths.

All data sets were split into a training set, and a validation set at a ratio of 8:2. To eliminate the randomness of the experiments, all experiments were repeated ten times to take the average. The STSGCN model was designed using Python in the MXNet [43] library, and the model parametric learning rate is set to 0.001, batch size is set to 32, and epochs are set to 3000. The STSGCN model consists of four STSGCLs, each STSGCM contains 3 graph convolution operations with 64, 64, and 64 filters. The input time series length has a significant impact on the traffic flow prediction. To further analyze the effect of input historical time series length on the prediction performance, the historical time series input length is tested from 1 to 16. Fig 7 shows the trend of RMSE with increasing input series length, and the blue line shows the trend of MAE. From the figure, we can know that a historical input sequence length of 12 is the best. Therefore, we use the past 12 consecutive time intervals to predict the traffic flow for the next time interval.

### Experimental results and analysis

**Fig 8:**
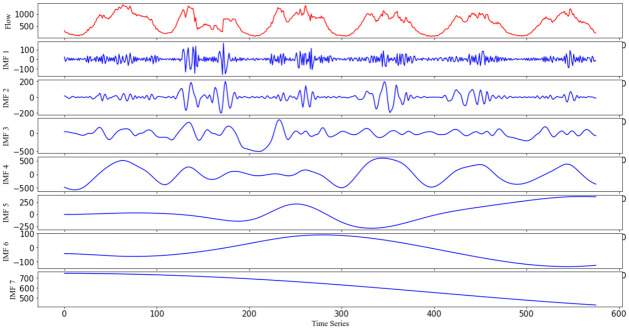
Decomposition results of the traffic flow after CEEMDAN.

Before inputting the data into the model, we first decompose the original non-stationary characteristics traffic flow signal by CEEMDAN, and in the decomposition process, we add 500 groups of white noise signals with a standard deviation of 0.2. The decomposition results are shown in Fig 8, in which the original time series of traffic is decomposed into 7 IMF components with different randomness, and the adjacent IMF components fluctuate to a similar degree.

To analyze the predictive performance of our proposed model, we use ETC gantry data and toll data to compare the following methods, which include regression models based on mathematical statistics, machine learning models and deep learning models.

Autoregressive Integrated Moving Average mode (ARIMA): ARIMA is widely used in traffic flow forecasting as a time series forecasting method based on mathematical and statistical models.

Support Vector Regression (SVR): SVR model applies linear support vector machines for regression analysis.

Gate Recurrent Unit (GRU): Used for time series prediction.

Graph Convolutional Network (GCN): Well known for its excellent performance in capturing spatial correlation and widely used for capturing time series data processing.

STDN [44]: Proposes a low-gated local CNN for spatial modeling of dynamic similarity between locations, and LSTM for handling long-term periodic information and temporal variation in a layered manner.

STGCN: Consists of a graph convolution layer and a convolutional sequence learning layer to model spatial and temporal dependencies, respectively.

ASTGCN: Attention-based spatial-temporal graph convolutional networks are designed to capture complex dynamic spatial-temporal correlations with spatial attention mechanism and temporal attention mechanism, respectively.

STSGCN: Merges three-time steps into one graph to capture spatial dependencies and temporal correlations simultaneously.

#### Prediction performance comparison of key road sections

During the holidays, not all expressway sections have significant holiday characteristics. Traffic flows in sections adjacent to key cities may have obvious holiday characteristics, while those undertaking daily commuting or suburban sections have no obvious characteristics.

In this paper, according to the document “Information and Distribution Map of 32 Congestion-prone Sections on Fujian Expressway” published by Fujian Expressway Information Technology Co., Ltd, we selected congestion-prone road sections in different areas on the Fuzhou-Xiamen Expressway to show the prediction performance of our proposed method. Those four sections are Xinglin Interchange Section, Xiang’an Interchange Section, Chidian Interchange Section, and Putian Interchange Section. Tables 4 and 5 shows the prediction performance of our model compared with other baseline models.

**Table 4:** Comparison of prediction results between Xinglin Interchange Section and Xiang’an Interchange Section.

Models	Xinglin Interchange Section			Xiang’an Interchange Section		
	RMSE	MAE	MAPE(%)	RMSE	MAE	MAPE(%)
ARIMA	64.893	40.524	23.81	65.653	41.293	24.95
SVR	38.512	25.047	19.29	39.643	25.951	19.85
GCN	33.532	23.548	18.45	33.645	23.718	18.73
GRU	32.466	22.624	17.92	32.866	22.716	18.16
STDN	30.871	20.148	16.78	30.975	20.568	17.35
T-GCN	30.534	18.972	16.57	30.549	19.372	16.76
STGCN	29.894	18.288	15.74	30.381	18.751	16.27
ASTGCN	29.325	16.853	15.38	29.855	16.693	15.92
STSGCN	28.614	16.688	14.19	28.749	16.695	14.62
**Our Model**	**26.428**	**15.235**	**13.31**	**26.598**	**15.295**	**13.47**

**Table 5:** Comparison of prediction results between Chidian Interchange Section and Putian Interchange Section.

Models	Chidian Interchange Section			Putian Interchange Section		
	RMSE	MAE	MAPE(%)	RMSE	MAE	MAPE(%)
ARIMA	65.923	41.724	25.26	65.748	41.459	25.07
SVR	39.852	26.047	19.92	39.715	25.972	19.83
GCN	33.894	23.876	18.83	33.728	23.784	18.82
GRU	32.956	22.741	18.26	32.903	22.728	18.21
STDN	31.951	20.698	17.52	31.428	20.624	17.42
T-GCN	31.537	19.573	16.91	31.154	19.427	16.81
STGCN	30.584	18.968	16.32	30.462	18.851	16.29
ASTGCN	29.982	17.326	16.25	29.906	17.106	16.12
STSGCN	29.214	16.988	14.92	28.985	16.782	14.75
**Our Model**	**27.128**	**15.965**	**13.84**	**26.858**	**15.576**	**13.64**

Among these four road sections, ARIMA performs the worst regardless of the section, with RMSE, MAE, and MAPE of 64.893, 40.524, and 0.2381, respectively. The reason is that ARIMA has great limitations in capturing complex traffic flow data and can only capture a certain amount of temporal correlation. In addition, we compare our model with other machine learning and deep learning prediction models. SVR can only capture a limited range of nonlinear features, so its prediction performance is only better than ARIMA. GCN can capture more spatial correlation, and GRU can capture more temporal correlation, so their performance is better than SVR. T-GCN, STDN, STGCN, and ASTGCN simultaneously consider spatial-temporal features, thus achieving improved prediction accuracy. However, these models are predicted for general weekday traffic flows, therefore, they are not suitable for application to predict holiday traffic flows. We propose a deep learning-based traffic flow prediction model for holidays.

To better explore the holiday characteristics of holiday traffic flows, we consider the effects of the quality and quantity of traffic flow data, for improving the accuracy of holiday traffic flow prediction. In addition, the CEEMDAN algorithm has the ability to solve the uncertainty of traffic flow, STSGCN has the ability to powerfully capture local spatial-temporal correlation and topological information, and the FCM algorithm can solve the problem of lack of traffic flow data during holidays. On this basis, we propose a holiday traffic flow prediction model. The combined model CEEMDAN-STSGCN-FCM proposed achieves the best prediction performance with RMSE, MAE, and MAPE of 26.428, 15.235, and 0.1331, respectively.

**Fig 9:**
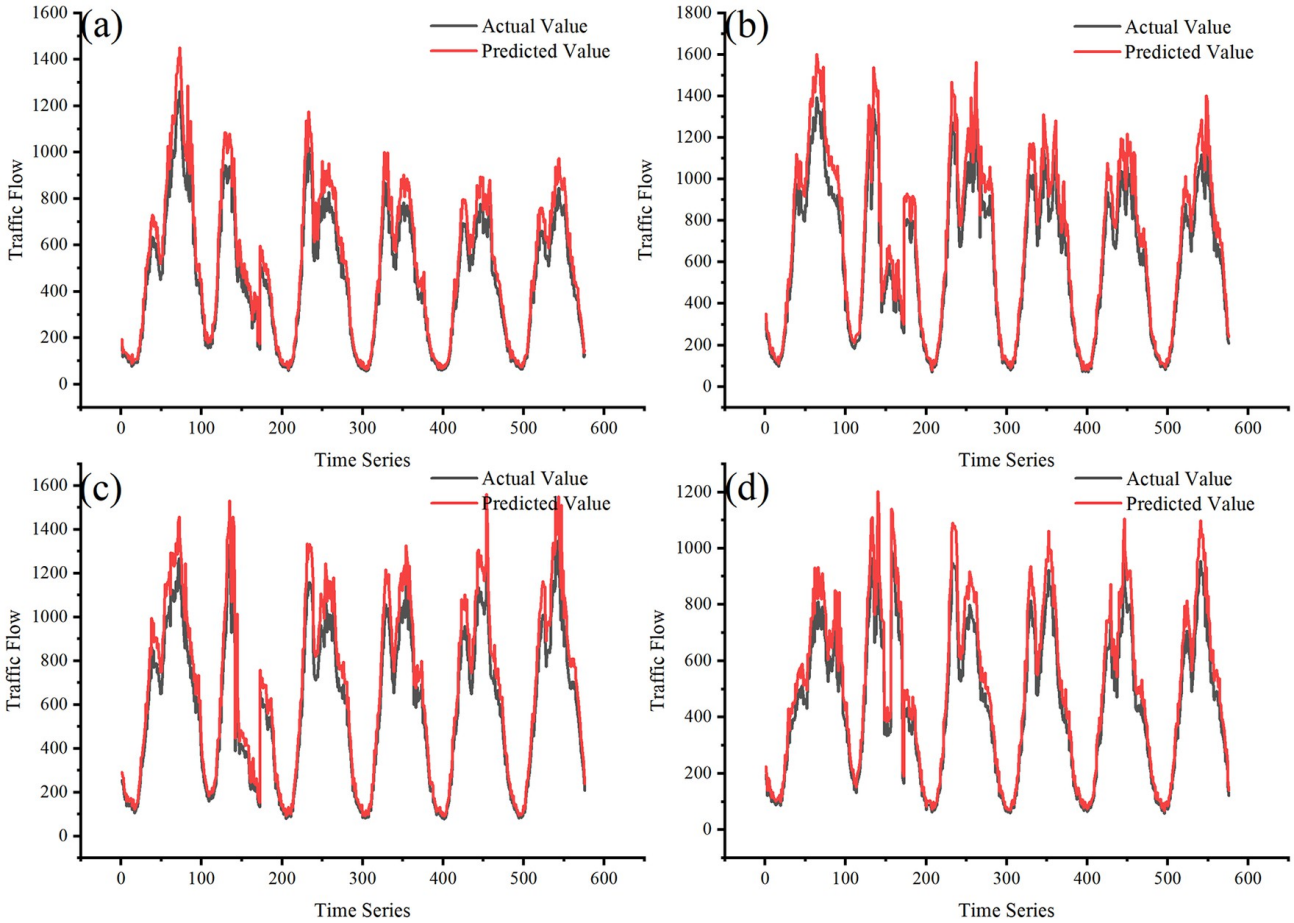
Comparison of the prediction performance for different models. (a) Xinglin Interchange Section; (b) Xiang’an Interchange Section; (c) Chidian Interchange Section; (d) Putian Interchange Section.

Fig 9 shows the predicted results for the Xinglin Interchange Section, the Xiang’an Interchange Section, the Chidian Interchange Section, and the Putian interchange section, respectively. It can be seen that the traffic flow shows obvious holiday characteristics: the peak is more significant than the usual. Our proposed model can fully capture the spatial-temporal characteristics of different types of traffic flows, and its prediction results closely match with the actual values. Therefore, the CEEMDAN-STSGCN-FCM model has strong robustness and can make accurate traffic flow predictions for different types of key sections.

#### Prediction performance for different time periods

**Fig 10:**
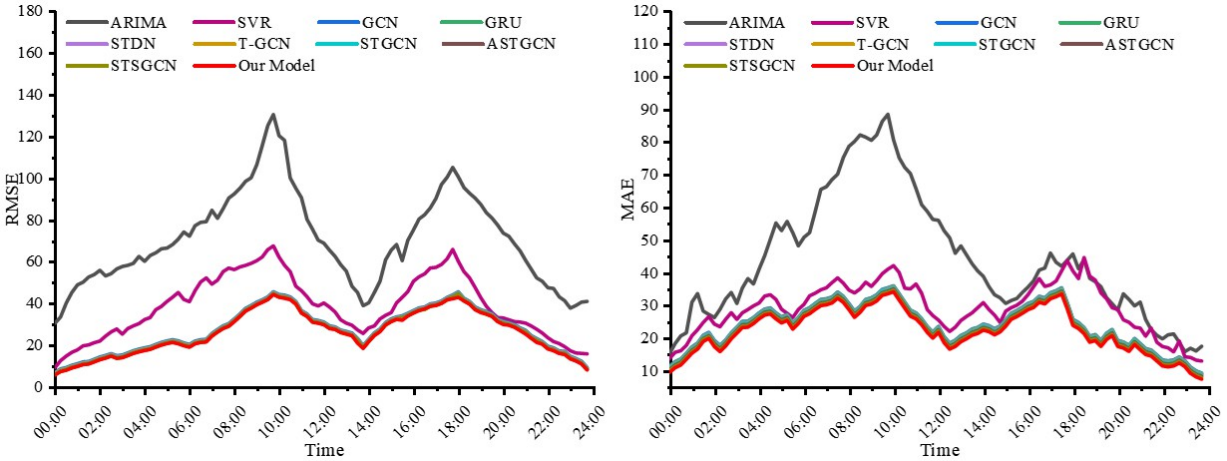
Performance comparison of models for different time periods on the Xinglin interchange section.

In order to explore in depth the prediction performance of CEEMDAN-STSGCN-FCM for different time periods, the average loss of the Xinglin interchange section was analyzed. Fig 10 shows the prediction performance of our proposed model with other baseline models for different time periods. When the traffic flow increases sharply, the performance indicators increase as well. The performance metrics of the statistics-based model ARIMA have the most significant peak characteristics, while the evaluation metrics of our CEEMDAN-STSGCN-FCM model exhibit the smallest peak characteristics in all periods. The results show that the CEEMDAN-STSGCN-FCM exhibits better stability and robustness in the peak and off-peak periods, and is able to achieve better forecasting results during holidays.

#### Prediction performance of different components

To further explore the impact of different components on prediction accuracy in our model architecture, we studied the construction of each component. First, we removed the CEEMDAN component or FCM component and kept the others unchanged in structure to show their effects on the prediction performance. Then, we compared various modules in the STSGCN model, for example, without setting the spatial-temporal embedding matrix and the mask matrix. Finally, we compared the RMSE, MAE, and MAPE of the prediction results.

1. Without CEEMDAN: Remove CEEMDAN from CEEMDAN-STSGCN-FCM.
2. Without FCM: Remove FCM from CEEMDAN-STSGCN-FCM.
3. Without mask: The mask matrix does not exist in the STSGCN model.
4. Without emb: The spatial-temporal embedding matrix is not added in STSGCL.

**Fig 11:**
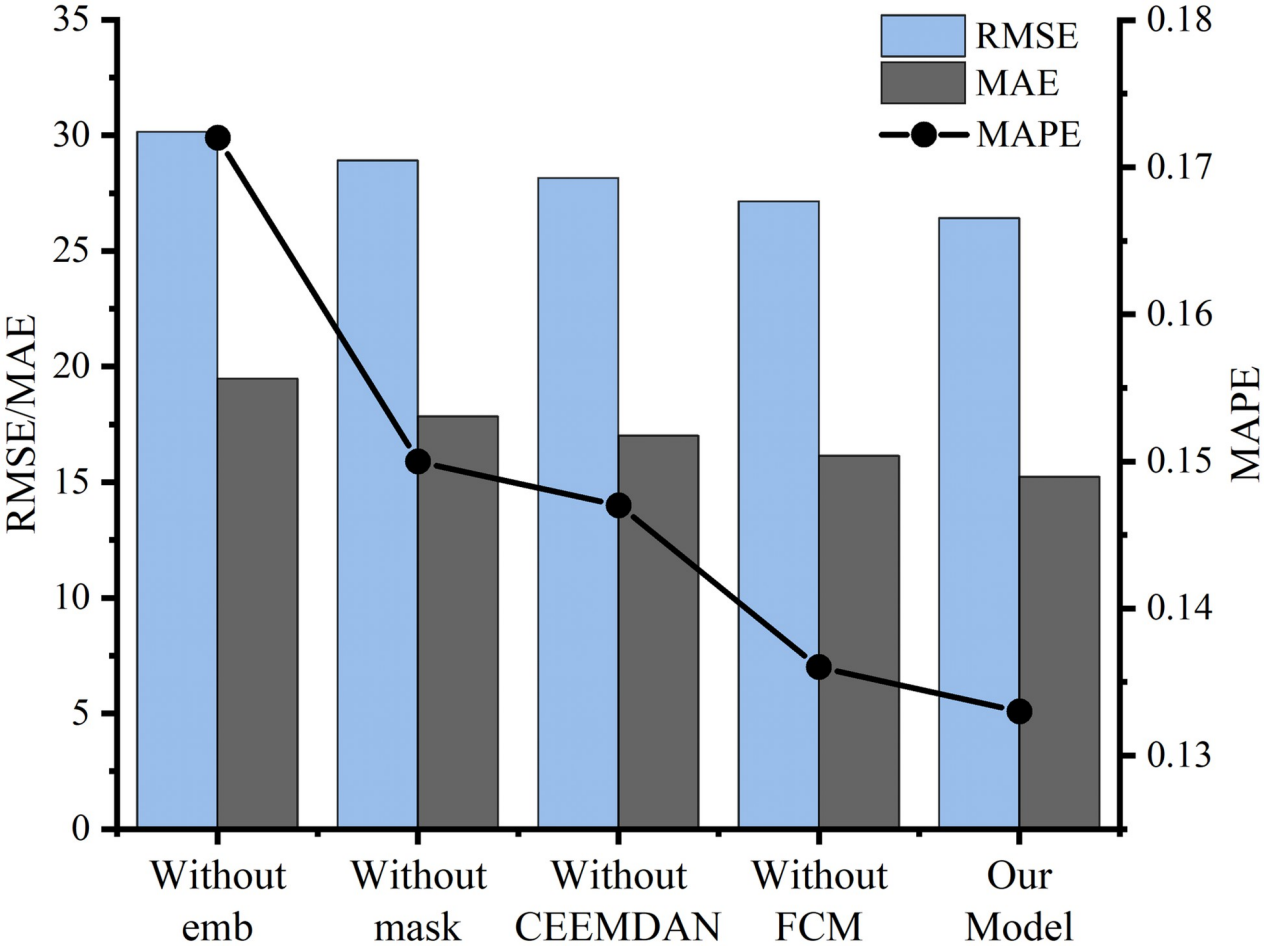
Prediction performance of different components.

Table 6 and Fig 11 illustrate the experimental results of the different compositional research. According to the results, we conclude that the CEEMDAN-STSGCN-FCM outperforms the other conditions with the lowest RMSE of 26.428, the lowest MAE of 15.235; the lowest MAPE of 0.133.

**Table 6:** Comparison of prediction performance of different components.

Model	RMSE	MAE	MAPE
Without emb	30.155	19.481	0.172
Without mask	28.9241	17.843	0.15
Without CEEMDAN	28.156	17.013	0.147
Without FCM	27.149	16.148e	0.136
**CEEMDAN-STSGCN-FCM**	**26.428**	**15.235**	**0.133**

CEEMDAN-STSGCN-FCM without spatial-temporal embedding matrix performs the worst when various constituents are analyzed, indicating that the spatial-temporal embedding matrix in STSGCN can adequately capture the spatial-temporal characteristics of traffic flow and thus effectively improve the prediction performance. The prediction accuracy is significantly improved by adding a mask matrix to adjust the weights between each node in the graph convolution. The prediction performance of the model decreases when the model does not have CEEMDAN, indicating that CEEMDAN can improve the prediction performance because it enhances the ability to capture the nonlinear and nonsmooth signal features. The prediction accuracy of CEEMDAN-STSGCN is low when the FCM algorithm is not used, while the performance is significantly improved after using the FCM algorithm, indicating that the FCM algorithm can better reflect the characteristics of holiday traffic flow and effectively overcome the problem of inadequate prediction accuracy because of the limited sample size of holiday traffic flow. The above results show that our model architecture can fully capture the spatial and temporal characteristics of traffic flow on key sections during holidays, thus ensuring a better prediction of holiday traffic flow.

## Conclusion

During holidays, due to the suddenness and irregularity of expressway traffic flow, forecasting traffic flow during holidays is a difficult task for traffic management. We propose a CEEMDAN-STSGCN-FCM model based on ETC gantry data and toll data for short-term traffic flow prediction during holidays. The main conclusions are as follows.

1. The proposed CEEMDAN-STSGCN-FCM has obvious advantages in capturing the spatial-temporal correlation of traffic flow, especially in the expressway road network on holidays.
2. Our proposed method can not only efficiently capture the spatial-temporal correlation of vehicles in route, but also can consider the spatial-temporal correlation of vehicles that will be proceeding from toll stations.
3. The results of testing on real ETC gantry data and toll data show that CEEMDAN-STSGCN-FCM performs well under different studies and shows good robustness.

However, there are some limitations to our study. For example, the effects of other factors on traffic flow are not considered. In the next step, we will consider multiple data sources, such as COVID-19 data, weather data, to improve prediction accuracy based on real-time changes in data.
